# Effects of corticosterone on the metabolic activity of cultured chicken chondrocytes

**DOI:** 10.1186/s12917-015-0398-5

**Published:** 2015-04-08

**Authors:** Hua Zhang, Zhenlei Zhou, Jingwen Luo, Jiafa Hou

**Affiliations:** College of Veterinary Medicine, Nanjing Agricultural University, Nanjing, Jiangsu 210095 China

**Keywords:** Corticosterone, Chondrocytes, ALP, Col X, PTHrP

## Abstract

**Background:**

Corticosterone is one of the most crucial glucocorticoids (GCs) in poultry. Our previous study shows that corticosterone can retard the longitudinal growth of bones by depressing the proliferation and differentiation of chondrocytes in broilers. The present study was designed to investigate whether corticosterone affect the development of chondrocytes and the synthesis of collagen *in vitro*. The chondrocytes were isolated from proximal tibial growth plates of 6-week-old broiler chickens and cultured with different doses of corticosterone for 48 h. Then the cell viability, alkaline phosphatase (ALP) activity and the expression of parathyroid hormone-related peptide (PTHrP) and type X collagen (Col X) were detected.

**Results:**

At 10^−9^-10^−6^ M concentration, corticosterone significantly inhibited the viability and differentiation of chondrocytes, as indicated by decreases in ALP and type X collagen expression. Conversely, there was completely opposite effect at 10^−10^ M. In addition, the expression of PTHrP was significantly downregulated at 10^−6^ M and 10^−8^ M, and was upregulated at 10^−10^ M.

**Conclusions:**

The results suggested that corticosterone regulated chicken chondrocytes performance depending on its concentration with high concentrations inhibiting the viability and differentiation of chondrocytes and light concentrations promoting them, and these roles of corticosterone may be in part mediated through PTHrP.

**Electronic supplementary material:**

The online version of this article (doi:10.1186/s12917-015-0398-5) contains supplementary material, which is available to authorized users.

## Background

Longitudinal bone growth depends on the proliferation, differentiation of growth plate chondrocytes by complex endocrine regulation [1]. It is well known that glucocorticoids (GCs) have complex effects on bone metablism. Prolonged or high dose glucocorticoid administration as well as an excess of endogenous production of GCs increases the risk of bone disorders [2]. Corticosterone (CORT) is the main glucocorticoid hormone in birds [3] and in charge of bone metabolism [2]. Numerous studies had been conducted concerning the effects of GCs on growth retardation in mammals [4-15] and were to some extent controversial [16,17], whereas relatively few data were available with regard to its roles in bone development in avian.

It was reported that parathyroid hormone-related peptide (PTHrP) was one of the important cytokines. Both the periarticular perichondrium and growth plate chondrocytes are the source of PTHrP [18,19]. Ablation of the PTHrP gene exhibits a delay in chondrocyte differentiation and leads to distinct abnormalities in bone development [20]. PTHrP directly stimulates chondrocyte proliferation and prevents the differentiation of proliferating chondrocytes to prehypertrophic cells [21,22]. Type X collagen (Col X) expressing exclusively in the matrix of the hypertrophic zone [23,24] is one of the well-known markers of chondrocyte hypertrophy [25,26].

Previous studies from our laboratory showed that exposure to CORT depressed the longitudinal growth of the long bones by inhibiting the proliferation and differentiation of chondrocytes in growth plate in broilers [27], but the effects of CORT on the cellular events that occurred during this process in birds were uncertain, the present study was designed to investigate whether CORT affect the performance of chondrocytes obtained from the epiphyseal growth plates of broiler chickens by determining the ALP activity and expression of PTHrP and ColX.

## Results

### Effect of CORT on cell viability and ALP activity in cultured chicken chondrocytes

**Figure 1:**
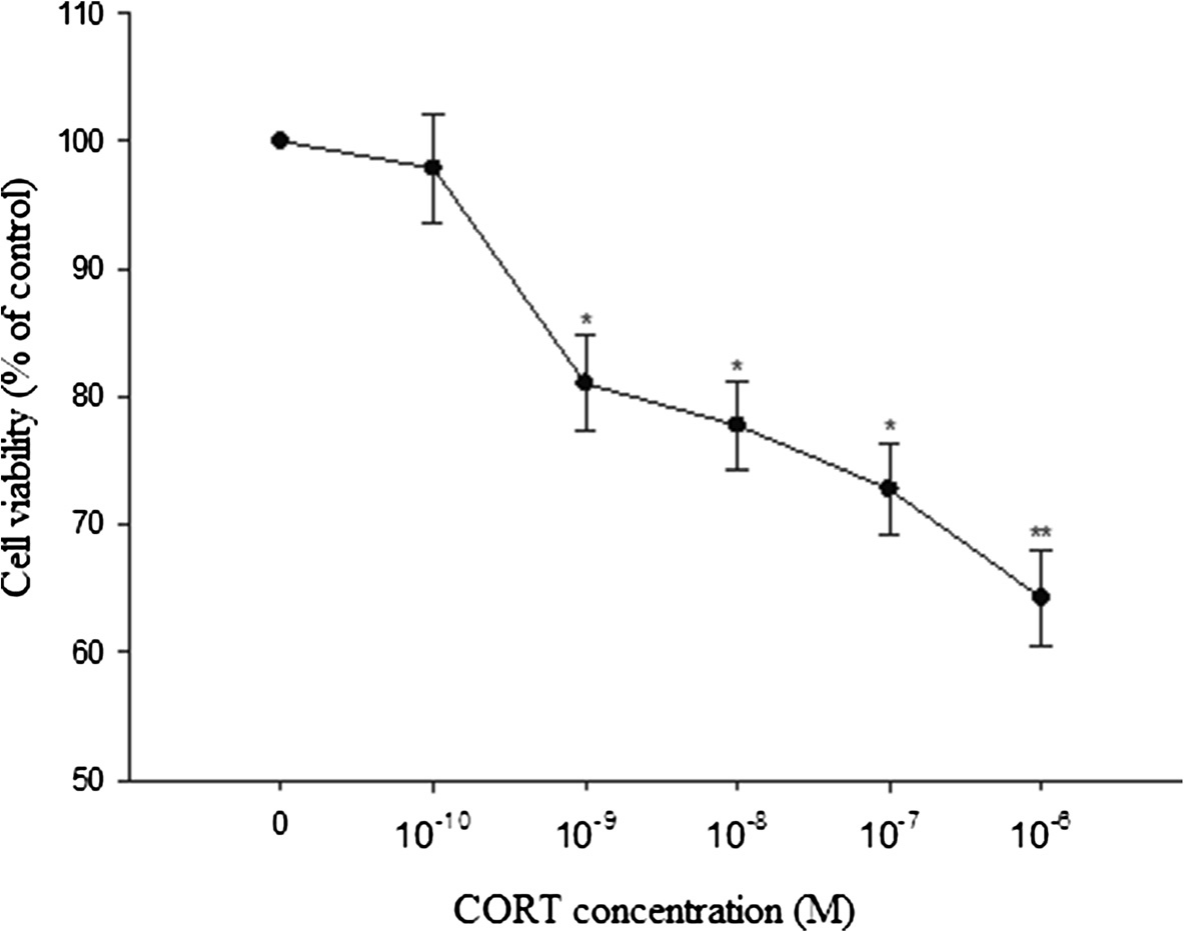
**Effects of various doses CORT on cell viability measured by MTT assay.** The cells were incubated with increasing concentrations of CORT for 48 h. Treated cell viabilities were expressed as a percentage of control (100%). Data were mean ± SEM from at least three separate experiments, each performed in triplicates. *P < 0.05 and **P < 0.01 versus control (0 M CORT).

**Figure 2:**
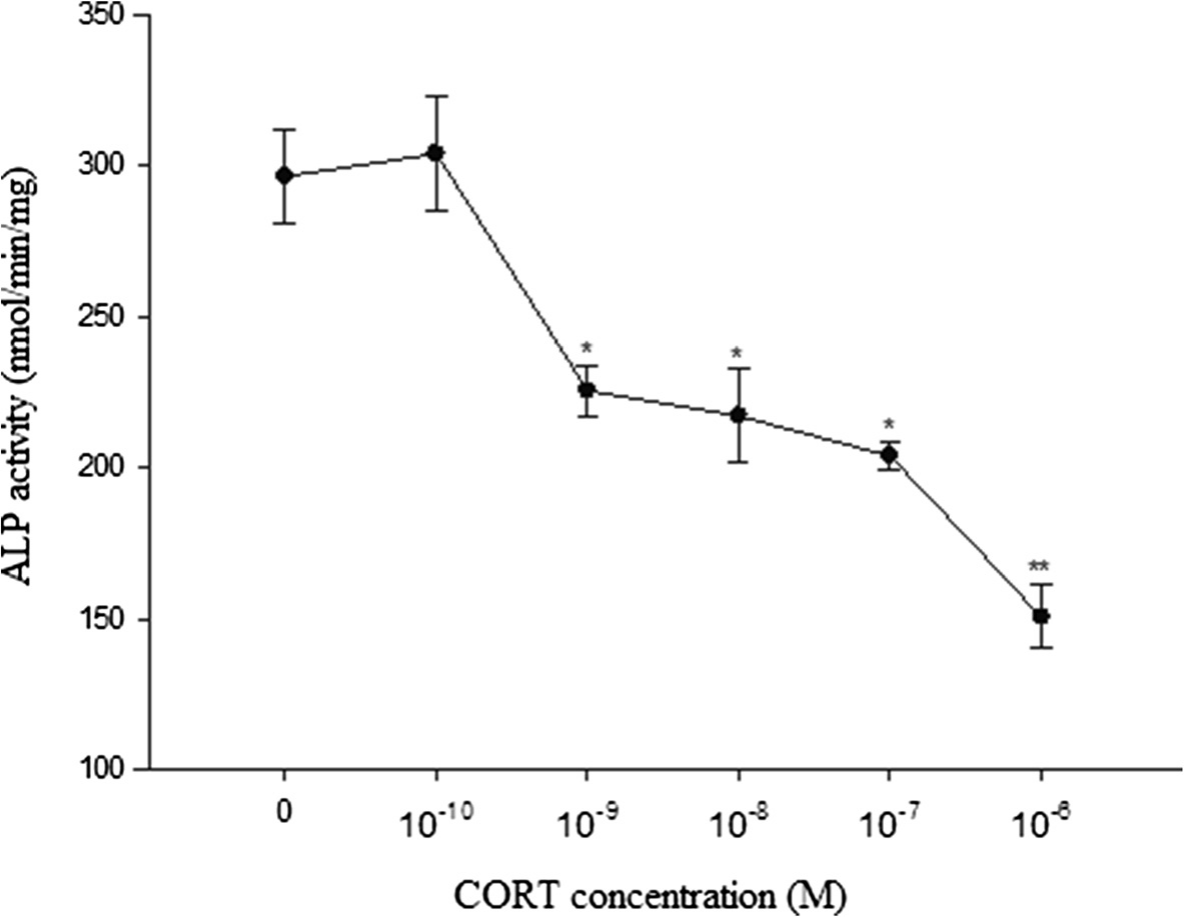
**Effects of various doses CORT on intracellular ALP activity.** The cells were incubated with increasing concentrations of CORT for 48 h. Values were mean ± SEM from at least three separate experiments, each performed in triplicates. *P < 0.05 and **P < 0.01 versus control (0 M CORT).

The results showed that CORT inhibited chondrocytes viability and ALP activity significantly at concentrations of 10^−9^ M to 10^−6^ M in a dose-dependent manner (Figures 1 and 2). The lowest chondrocytes viability was observed at 10^−6^ M CORT reducing the cell viability as great as to 64.2% of the control. And the lowest ALP activity (150.57 ± 10.591 nmol/min/mg) was also measured at 10^−6^ M CORT. However, 10^−10^ CORT administration did not affect chondrocytes viability and ALP activity. Additional tables file show this in more detail [see Additional files 1 and 2]. Based on these results, 10^−10^ M, 10^−8^ M and 10^−6^ M CORT (as low, middle and high concentration, respectively) were determined to evaluate its roles in the regulation of gene expression in cultured growth plate chondrocytes.

### Effect of CORT on expression of PTHrP and ColX in cultured chicken chondrocytes

**Figure 3:**
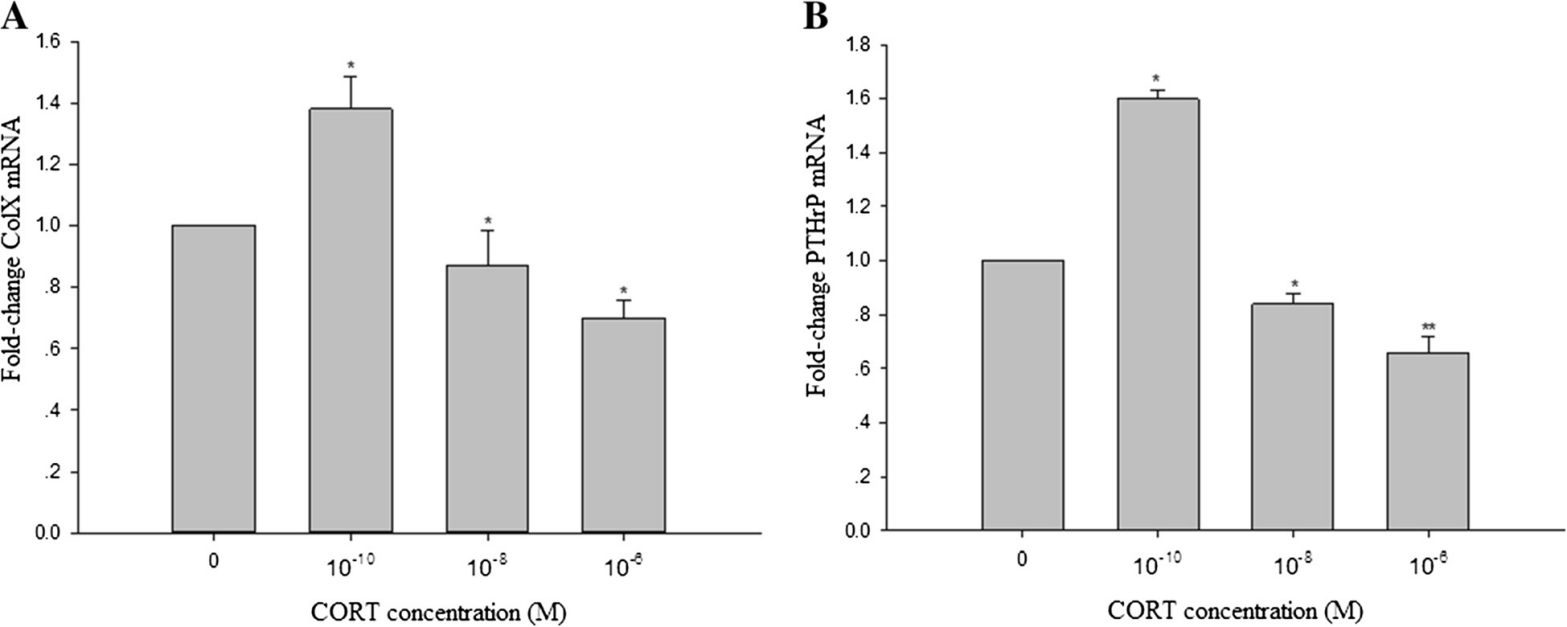
**Effects of CORT on PTHrP and Col X mRNAs expression in cultured Chondrocytes (A-B).** The cells were incubated with various doses of CORT for 48 h. The relative expression of PTHrP and Col X genes was analysed by real-time PCR. Gene expression was normalized using GAPDH as an internal control. Values were mean ± SEM from at least three separate experiments, each performed in triplicates. *P < 0.05 and **P < 0.01 versus control (0 M CORT).

**Figure 4:**
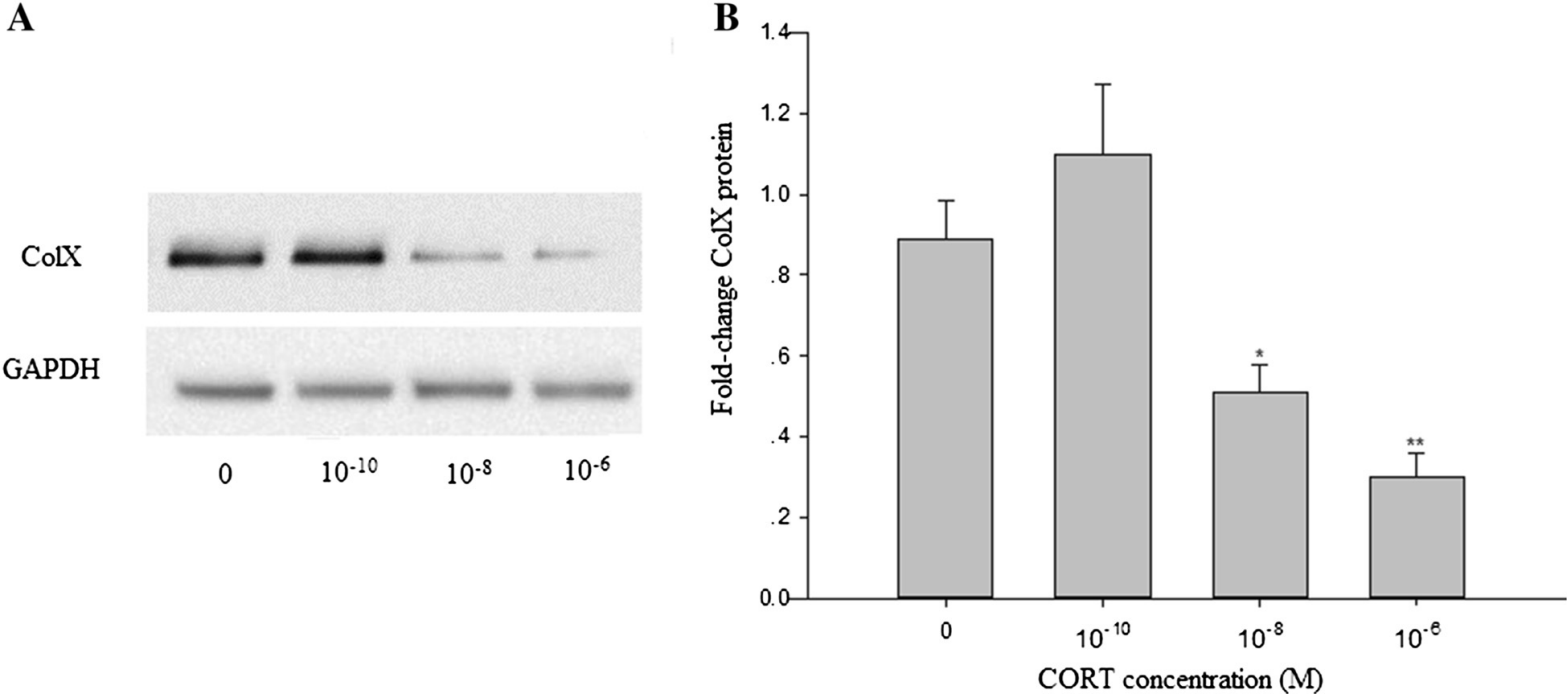
**Western blot for Col X from cultured chondrocytes exposed by different dose of CORT. (A)** Immunoreactive bands for Col X and GAPDH protein. **(B)** Statistical analysis of Col X. Cultures were lysed and protein was extracted, quantitated and analyzed via Western blot analysis. Representative blot for each group from triplicate experiments was shown. Col X protein band density was quantitated by densitometry using the Quantity One software. Col X protein level was expressed as the Col X: Gapdh ratio. *P < 0.05 and **P < 0.01 versus control (0 M CORT).

Expression of related genes was investigated after 48 h exposure of different doses of CORT (10^−10^ M, 10^−8^ M and 10^−6^ M). Real-time RT-PCR showed that 10^−10^ M CORT resulted in obviously increasing the expression of PTHrP and Col X (Figure 3A and B). Furthermore, 10^−8^ M and 10^−6^ M CORT significantly declined PTHrP and Col X mRNA levels, and the inhibition exerted at the concentration of 10^−6^ M CORT was more effective. The Col X protein levels (expressed as the Col X: Gapdh ratio) displayed the same changes as its mRNA expression after CORT treatment (Figure 4).

## Discussion

In the present study, we investigated the effects of CORT on cultured chicken growth plate chondrocytes in terms of cell viability, ALP activity and the expression of Col X and PTHrP. Growth plate chondrocytes were exquisitely sensitive to GCs [6,27,28]. The results showed that PTHrP signaling could play a crucial role in regulating the viability and differentiation of growth plate chondrocytes of chickens.

Based on the present study, MTT assay was performed to evaluate the effect of CORT on the viability of chondrocytes for 48 h. Interestingly, CORT at the concentration of 10^−10^-10^−6^ M didn’t have a clear dose-dependent effect on cell viability. CORT could inhibit chondrocytes viability at the concentration of 10^−9^-10^−6^ M and have no effect with 10^−10^. M. R. Quarto et al. [17] suggested that GCs can support chondrocytes viability. However, it was difficult to compare our observations with other studies because the origin of the investigated chondrocytes, the culture conditions, the types and doses of GCs and the exposure times were distinct.

The process of endochondral ossification which consists of cartilage formation and replacement of cartilage by bone plays a major role in bone development [29]. The chondrocytes actively proliferate and synthesize a large amount of extracellular matrix components undergo a series of maturation events. They advance to the hypertrophic stage, which is characterized by the expression of type X collagen and ALP [30]. In the process of chondrocytes maturation, ALP secreted from chondrocytes could convert organic phosphorus esters to inorganic phosphates by hydrolysis, forming a hydroxyapatite precursor which then undergoes calcification [31]. Therefore, ALP is recommended as a marker of chondrocyte maturation [32]. In this study, we observed that the decrease in ALP activity was accompanied by a decline in the viability of choncrocytes after CORT treatment at 10^−9^ -10^−6^ M. It indicated that the supression of chondrocytes differentiation and the reduction of ALP activity may be due to inhibitory effect of CORT on the viability of chondrocytes.

Col X, which is a recognized marker of chondrocyte hypertrophy, is mainly synthesized by hypertrophic chondrocytes and plays a key role in endochondral ossification. The current study showed that the expression of Col X was inhibited by CORT with middle and high concentration, while the expression of Col X was significantly increased with low concentration CORT treatment. These results provided an evidence for our previous study in vivo that excess CORT inhibited the growth of proliferative and prehypertrophic chondrocytes and the synthesis of Col X [27]. This finding, together with the variation of ALP activity and cell viability, clearly indicated that CORT not only affected cell viability, but also inhibited the differentiation process of growth plate chondrocytes.

A number of growth factors are known to be regulators of chondrocytes development. PTHrP plays important roles in longitudinal bone growth, as it is a negative regulator of growth plate chondrocytes terminal differentiation [18]. As a key cytokine, PTHrP maintains chondrocytes in a proliferative state and regulate chondrocytes proliferation [33,34]. PTHrP blocks matrix calcification and decrease ALP activity markedly in chondrocytes through PKC/p38 signaling pathways [30]. The present study showed that CORT had distrinct impact on the expression of PTHrP mRNA with various concentrations. In addition, the changes of levers of Col X and ALP in chondrocytes were coordinated with trends of PTHrP expression following treatment with CORT. Our finding indicated that the regulation of the viability and differentiation of chondrocytes by CORT might be in part mediated through by PTHrP. More studies were still needed to further to determine whether there were interactions between CORT and PTHrP.

## Conclusions

Based on the outcome, our data confirmed that CORT had profound impacts upon bone growth through orderly process of differentiation of growth plate chondrocytes reported by our previous study but now amongst in vivo circumstances [27]. PTHrP may play a certain role in regulating chondrocytes growth induced by CORT. The effect of CORT may be in part mediated by the expression of PTHrP.

## Methods

### Chemicals and reagents

Dulbecco’s modified eagle’s medium (DMEM), penicillin-streptomycin solution, fetal bovine serum (FBS), amphotericin B solution, collagenase type IV and Trizol reagent were procured from Invitrogen, Carlsbad, CA, USA. Bovine testicular hyaluronidase, a nonessential amino acids mixture (100×) and CORT were purchased from Sigma-Aldrich, Shanghai, China. Two-step reverse-transcriptase polymerase chain reaction kit was purchased from TaKaRa Biotechnology (Dalian) Co., Ltd., China. Col X antibody, anti Gapdh mouse monoclonal antibody, HRP conjugated goat anti-rabbit IgG (H+L) and HRP conjugated goat anti-mouse IgG (H+L) were purchased from Otwo Biotech Co., Ltd., China. Cell lysis buffer for Western was procured from Beyotime Institute of Biotechnology, China. Other chemicals and reagents used in the present study were of analytical grade and were obtained from the Jiancheng Bioengineering Institute (Nanjing, China) unless otherwise indicated.

All animal work was performed according to the international animal welfare guidelines, and protocols were approved by Nanjing Agricultural University Animal Care Committee. Animal studies were conducted in accordance with the recommendations in the Guidelines for the Care and Use of Laboratory Animals of the Ministry of Science and Technology of the People’s Republic of China ([2006] 398).

### Cell culture and treatment

Growth plate cartilage was harvested from the proximal end of the tibia of 6-week-old hybrid broiler chickens and chondrocytes were isolated as previously described [35]. Briefly, tibial growth plate slices were treated in a digest solution (0.1% type IV collagenase, 0.1% hyaluronidase, 5% FBS) and incubated at 37°C overnight. The resulting crude cell preparations were further purified on lymphocyte separation medium (MP Biomedicals, LLC, California, USA), resupended and counting, then inoculated to multi-well plates containing complete medium with the density for10^6^ cell/cm^2^. Culture medium (10% FBS, 1% nonessential amino acids mixture) was changed every other day with a fresh supplement of ascorbic acid (50 μg/ml) from day 3 onward, for the duration of the experiments. To facilitate cell attachment, 4U/mL testicular hyaluronidase was added to the medium. After the attachment of cells, the growth medium was then removed and cells were exposed to different doses of CORT (10^−10^ - 10^−6^ M) using serum-free fresh medium at 37°C and 5% CO_2_ for 48 h. All culture media contained 100 IU/ml of penicillin, 100 μg/ml of streptomycin and 0.25 μg/ml of amphotericin B.

### MTT assay

Cell viability was assessed by the ability of mitochondrial dehydrogenases to oxidize the 3-(4, 5-dimethylthiazol-2-yl)-2, 5-diphenyltetrazolium bromide (MTT) to a purple formazan product [27]. After 48 h exposure, 20 μl MTT (5 mg/ml) was added into each well and incubated for 4 h at 37°C. Purple formazan crystals were solubilized in dimethyl sulfoxide for 10 min at 37°C. The absorbance at 570 nm was measured in a Bio-Rad microplate reader (Bio-Rad Laboratories, Hercules, CA) and results were expressed as a percentage of control.

### Alkaline phosphatase (ALP) activity

Cell layers were washed twice with cold phosphate buffered saline (PBS pH = 7.4). The cell suspension was homogenized and 50 μl of each sample was aliquoted into a 96-well plate in duplicate to assay ALP activity and protein content [36]. 150 μl of a buffer solution consisting of 10 mM p-nitrophenyl phosphate (pNPP), 0.2 mM MgCl_2_ and 1 mM diethanolamine was added to each sample [35]. After incubated at 37°C for 30 min and then 100 μl of 0.1 M NaOH was added to stop the reaction. The plates were read in a Bio-Rad microplate reader at 405 nm. The ALP activity was determined by comparing the experimental samples with standard solutions of p-nitrophenol and an appropriate blank. Protein concentrations of the samples were determined using the BCA assay kit (Wuhan Boster Company, Wuhan, China). Each sample was normalized by the total protein content per well to determine specific activity. Total ALP activity was expressed as nmoles pNPP hydrolysed/min/mg protein.

### Real-time RT-PCR

Total RNA was isolated from chondrocytes using Trizol reagent after 48 h exposure of different doses of CORT (10^−10^ M, 10^−8^ M and 10^−6^ M). Real-time RT-PCR was carried out using two-step RT-PCR kit per the instructions of the manufacturer. Gapdh, the house-keeping gene, was used as an internal control of the quantity and quality of the cDNAs. The primer sequences used for PCR amplification for Col X, PTHrP and Gapdh were listed in Table 1. Real-time PCR amplification was performed on LightCycler (Applied Biosystems, Inc., Foster City, CA). A Light Cycler melting curve was constructed to test for a single product at the end of each PCR reaction. Analysis of the relative gene expression level was achieved by using the 2^-ΔΔC^_T_ method for fold induction, and C_T_ (the threshold cycle) indicated the fractional cycle number at which the amount of amplified target reached a fixed threshold [37].

**Table 1:** **Sequence of primers used to amplify specific mRNA by real-time RT-PCR**

**Name**	**Primer sequence (5′-3′)**	**Product size (bp)**	**NCBI Genbank**
Col X^#^	Sense: AGTGCTGTCATTGATCTCATGGA	83	L11BB9.1
	Anti-sense: TCAGAGGAATAGAGACCATTGGATT		
PTHrP	Sense: CGGAGGATATGATGTTCAC	79	AB175678.1
	Anti-sense: TAGGAGGGCACAGAATAAC		
GAPDH	Sense: GAACATCATCCCAGCGTCCA	132	NM_204305.1
	Anti-sense: CGGCAGGTCAGGTCAACAAC		

^#^The primers of Col X was quoted from Reference [38].

### Western blot analysis

After 48 h exposure of CORT (10^−10^ M, 10^−8^ M and 10^−6^ M), chondrocyte lysates were prepared with RIPA Lysis buffer (Beyotime, Nanjing, China), and centrifuged at 12,000 g. Protein concentration was determined by using the BCA assay kit. Fifty micrograms of total protein (per lane) were resolved by sodium dodecyl sulphate-polyacrylamide gel electrophoresis (SDS-PAGE; 10% acrylamide) and transferred onto a nitrocellulose membrane. The membranes were blocked with 5% skimmed milk in Tris-buffered saline-Tween (TBS-T) for 2 hours and then probed with antibodies. The primary antibodies to Col X (diluted 1:200), and anti-Gapdh (diluted 1:5000) were used. HRP conjugated goat anti-rabbit or mouse IgG (H+L) (diluted 1:5000) was used as a secondary antibody. Visualization of immunoreactive proteins was achieved using the ECL Western blotting detection reagents (Millipore Corporation, Billerica, USA). Molecular weights of the immunoreactive proteins were determined against a protein marker ladder. Band density was quantitated using the Quantity One software (Bio-Rad Laboratories, Hercules, CA).

### Statistical analysis

Tests were carried out in triplicate and all experiments were performed a minimum of three times, and the mean and standard error of the mean (SEM) were determined. Statistical analyses were conducted using SPSS 11.0 for Windows (SPSS Inc., Chicago, IL, USA). Data were analyzed by one-way Analysis of variance (ANOVA) followed by Duncan’s multiple range tests to assess the significance between the control and experimental groups. Statistical significance was considered at the level of P < 0.05.

## Additional files

Additional file 1:
**Additional numerical data for effects of various doses CORT on cell viability measured by MTT assay.**
Additional file 2:
**Additional numerical data for effects of various doses CORT on intracellular ALP activity.**

## Abbreviations

ALP: Alkaline phosphatase
ANOVA: Analysis of variance
Col X: Type X collagen
CORT: Corticosterone
DMEM: Dulbecco’s modified eagle’s medium
FBS: Fetal bovine serum
GCs: Glucocorticoids
MTT: 3-(4, 5-dimethylthiazol-2-yl)-2, 5-diphenyltetrazolium bromide
PBS: phosphate buffered saline
PNPP: P-nitrophenyl phosphate
PTHrP: Parathyroid hormone-related protein
SDS-PAGE: Sodium dodecyl sulphate-polyacrylamide gel electrophoresis
SEM: Standard error of the mean
TBST: Tris-buffered saline-Tween

## References

[1] Nilsson O, Marino R, De Luca F, Phillip M, Baron J (2005). Endocrine regulation of the growth plate. Horm Res.

[2] Castro M, Elias LL, Conde P, Elias L, Moreira AC (2011). Physiology and pathophysi-ology of the HPA. Cushing’s Syndrome.

[3] Costantini D, Fanfani A, Dell’omo G (2008). Effects of corticosteroids on oxidative damage and circulating carotenoids in captive adult kestrels (Falco tinnunculus). J Comp Physiol B.

[4] Gafni RI, Weise M, Robrecht DT, Meyers JL, Barnes KM, De-Levi S (2001). Catch-up growth is associated with delayed senescence of the growth plate in rabbits. Pediatr Res.

[5] Miyazaki Y, Tsukazaki T, Hirota Y, Yonekura A, Osaki M, Shindo H (2000). Dexamethasone inhibition of TGF beta-induced cell growth and type II collagen mRNA expression through ERK-integrated AP-1 activity in cultured rat articular chondrocytes. Osteoarthr Cartil.

[6] Siebler T, Robson H, Shalet SM, Williams GR (2002). Dexamethasone inhibits and thyroid hormone promotes differentiation of mouse chondrogenic ATDC5 cells. Bone.

[7] Smink JJ, Buchholz IM, Hamers N, van Tilburg CM, Christis C, Sakkers RJB (2003). Short-term glucocorticoid treatment of piglets causes changes in growth plate morphology and angiogenesis. Osteoarthr Cartil.

[8] Rauch A, Seitz S, Baschant U, Schilling AF, Illing A, Stride B (2010). Glucocorticoids suppress bone formation by attenuating osteoblast differentiation via the monomeric glucocorticoid receptor. Cell Metab.

[9] Sanchez CP, He YZ (2002). Alterations in the growth plate cartilage of rats with renal failure receiving corticosteroid therapy. Bone.

[10] Hofbauer LC, Zeitz U, Schoppet M, Skalicky M, Schuler C, Stolina M (2009). Prevention of glucocorticoid-induced bone loss in mice by inhibition of RANKL. Arthritis Rheum.

[11] McLaughlin F, Mackintosh J, Hayes BP, Mclaren A, Uings IJ, Salmon P (2002). Glucocorticoid-induced osteopenia in the mouse as assessed by histomorphometry, microcomputed tomography, and biochemical markers. Bone.

[12] Blondelon D, Adolphe M, Zizine L, Lechat P (1980). Evidence for glucocorticoid receptors in cultured rabbit articular chondrocytes. FEBS Lett.

[13] Ranz FB, Aceitero J, Gaytan F (1987). Morphometric study of cartilage dynamics in the chick embryo tibia. II. Dexamethasone-treated embryos. J Anat.

[14] Guerne PA, Desgeorges A, Jaspar JM, Relic B, Peter R, Hoffmeyer P (1999). Effects of IL-6 and its soluble receptor on proteoglycan synthesis and NO release by human articular chondrocytes: comparison with IL-1. Modulation by dexamethasone. Matrix Biol.

[15] Abbadia Z, Amiral J, Trzeciak MC, Delmas PD, Clezardin P (1993). The growth-supportive effect of thrombospondin (TSP1) and the expression of TSP1 by human MG-63 osteoblastic cells are both inhibited by dexamethasone. FEBS Lett.

[16] Kato Y, Gospodarowicz D (1985). Stimulation by glucocorticoid of the synthesis of cartilage-matrix proteoglycans produced by rabbit costal chondrocytes in vitro. J Biol Chem.

[17] Quarto R, Campanile G, Cancedda R, Dozin B (1992). Thyroid hormone, insulin, and glucocorticoids are sufficient to support chondrocyte differentiation to hypertrophy: a serum-free analysis. J Cell Biol.

[18] Farquharson C, Seawright E, Jefferies D (2001). Parathyroid hormone-related peptide expression in tibial dyschondroplasia. Avian Pathol.

[19] Medill NJ, Praul CA, Ford BC, Leach RM (2001). Parathyroid hormone-related peptide expression in the epiphyseal growth plate of the juvenile chicken: evidence for the origin of the parathyroid hormone-related peptide found in the epiphyseal growth plate. J Cell Biochem.

[20] Lanske B, Amling M, Neff L, Guiducci J, Baron R, Kronenberg HM (1999). Ablation of the PTHrP gene or the PTH/PTHrP receptor gene leads to distinct abnormalities in bone development. J Clin Invest.

[21] Kronenberg HM, Lanske B, Kovacs CS, Chung UI, Lee K, Segre GV (1998). Functional analysis of the PTH/PTHrP network of ligands and receptors. Recent Prog Horm Res.

[22] Lanske B, Karaplis AC, Lee K, Luz A, Vortkamp A, Pirro A (1996). PTH/PTHrP receptor in early development and Indian hedgehog-regulated bone growth. Science.

[23] Shen G (2005). The role of type X collagen in facilitating and regulating endochondral ossification of articular cartilage. Orthod Craniofac Res.

[24] Kwan AP, Cummings CE, Chapman JA, Grant ME (1991). Macromolecular organization of chicken type X collagen in vitro. J Cell Biol.

[25] Cancedda R, Cancedda FD, Castagnola P (1995). Chondrocyte differentiation. Int Rev Cytol Survey Cell Biol.

[26] Linsenmayer TF, Long F, Nurminskaya M, Chen Q, Schmid TM (1998). Type X collagen and other up-regulated components of the avian hypertrophic cartilage program. Prog Nucleic Acid Res Mol Biol.

[27] Luo JW, Zhou ZL, Zhang H, Ma RS, Hou JF (2013). Bone response of broiler chickens (Gallus gallus domesticus) induced by corticosterone. Comp Biochem Physiol A Mol Integr Physiol.

[28] Silvestrini G, Ballanti P, Patacchioli FR, Mocetti P, Di Grezia R, Wedard BM (2000). Evaluation of apoptosis and the glucocorticoid receptor in the cartilage growth plate and metaphyseal bone cells of rats after high-dose treatment with corticosterone. Bone.

[29] Ma RS, Zhou ZL, Luo JW, Zhang H, Hou JF (2013). The Ihh signal is essential for regulating proliferation and hypertrophy of cultured chicken chondrocytes. Comp Physiol B-Biochem Mol Biol.

[30] Iwamoto M, Kitagaki J, Tamamura Y, Gentili C, Koyama E, Enomoto H (2003). Runx2 expression and action in chondrocytes are regulated by retinoid signaling and parathyroid hormone-related peptide (PTHrP). Osteoarthritis Cartilage.

[31] Sanchez C, Deberg MA, Piccardi N, Msika P, Reginster JY, Henrotin YE (2005). Subchondral bone osteoblasts induce phenotypic changes in human osteoarthritic chondrocytes. Osteoarthritis Cartilage.

[32] Henson FM, Davies ME, Skepper JN, Jeffcott LB (1995). Localisation of alkaline phosphatase in equine growth cartilage. J Anat.

[33] Harrington EK, Lunsford LE, Svoboda KKH (2004). Chondrocyte terminal differentiation, apoptosis, and type X collagen expression are downregulated by parathyroid hormone. Anat Rec Part Discov Mol Cell Evol Biol.

[34] Kobayashi T, Chung UI, Schipani E, Starbuck M, Karsenty G, Katagiri T (2002). PTHrP and Indian hedgehog control differentiation of growth plate chondrocytes at multiple steps. Development.

[35] He SJ, Hou JF, Dai YY, Zhou ZL, Deng YF (2012). N-acetyl-cysteine protects chicken growth plate chondrocytes from T-2 toxin-induced oxidative stress. J Appl Toxicol.

[36] Kinney RC, Schwartz Z, Week K, Lotz MK, Boyan BD (2005). Human articular chondrocytes exhibit sexual dimorphism in their responses to 17 beta-estradiol. Osteoarthr Cartil.

[37] Livak KJ, Schmittgen TD (2001). Analysis of relative gene expression data using real-time quantitative PCR and the 2 (−Delta Delta C (T)) Method. Methods.

[38] Wang W, Xu JP, Kirsch T (2003). Annexin-mediated Ca2+ influx regulates growth plate chondrocyte maturation and apoptosis. J Biol Chem.

