# Supplementary material for: Effects of corticosterone on the metabolic activity of cultured chicken chondrocytes
Source: BMC Vet Res. 2015 Apr 8;11:86. doi: 10.1186/s12917-015-0398-5 (PMC4393584; doi:10.1186/s12917-015-0398-5)
Supplement: Additional file 1: — Additional numerical data for effects of various doses CORT on cell viability measured by MTT assay. [file 12917_2015_398_MOESM1_ESM.doc]

Additional file 1 –Effects of various doses CORT on cell viability measured by MTT assay.

| CORT concentration(M) | Cell viability |
| --- | --- |
| 0 | 100 |
| 10^-10^ | 97.9±4.305 |
| 10^-9^ | 81.04±3.715^*^ |
| 10^-8^ | 77.73±3.406^*^ |
| 10^-7^ | 72.77±3.515^*^ |
| 10^-6^ | 64.21±3.774^**^ |

The cells were incubated with increasing concentrations of CORT for 48h. Treated cell viabilities were expressed as a percentage of control (100%). Data were mean ± SEM from at least three separate experiments, each performed in triplicates. *P < 0.05 and **P < 0.01 versus control (0 M CORT)
