# Supplementary material for: Effects of corticosterone on the metabolic activity of cultured chicken chondrocytes
Source: BMC Vet Res. 2015 Apr 8;11:86. doi: 10.1186/s12917-015-0398-5 (PMC4393584; doi:10.1186/s12917-015-0398-5)
Supplement: Additional file 2: — Additional numerical data for effects of various doses CORT on intracellular ALP activity. [file 12917_2015_398_MOESM2_ESM.doc]

Additional file 2-Effects of various doses CORT on intracellular ALP activity

| CORT concentration(M) | ALP viability(nmol/min/mg) |
| --- | --- |
| 0 | 296.36±15.368 |
| 10^-10^ | 303.88±18.552 |
| 10^-9^ | 225.5±8.356^*^ |
| 10^-8^ | 217.06±15.45^*^ |
| 10^-7^ | 203.81±4.502^*^ |
| 10^-6^ | 150.57±10.591^**^ |

The cells were incubated with increasing concentrations of CORT for 48h. Values were mean ± SEM from at least three separate experiments, each performed in triplicates. *P < 0.05 and **P < 0.01 versus control (0 M CORT).
